# Music Interventions in Hyperacute and Acute Stroke Patients: A Randomized Controlled Pilot Feasibility Study

**DOI:** 10.1002/acn3.70024

**Published:** 2025-03-03

**Authors:** Jeffrey J. Fletcher, Allison Edberg, Ronald Grifka, Joan Westendorp, Augusto Elias, Jacquie Knott, Elizabeth Martin, Fazeel Siddiqui

**Affiliations:** ^1^ Department of Neurosciences University of Michigan Health‐West Wyoming Michigan USA; ^2^ Department of Emergency Medicine University of Michigan Health‐West Wyoming Michigan USA; ^3^ Research Department University of Michigan Health‐West Wyoming Michigan USA; ^4^ Department of Nursing University of Michigan Health‐West Wyoming Michigan USA

**Keywords:** arterial pressure, complimentary therapies, music therapy, stroke

## Abstract

**Objective:**

Music interventions have been shown to have beneficial effects on hemodynamic parameters, pain, and anxiety in various medical settings. However, music interventions in the setting of acute stroke have not been studied. The objective of this trial was to perform a pilot feasibility study of music interventions in the setting of acute stroke to inform a larger efficacy trial.

**Methods:**

Open label parallel group, randomized controlled trial with objective endpoints.

**Results:**

The percentage of eligible patients approached for consent who were recruited into the trial was 85.7% (95% CI 75.9%–98%; 30/35) and the percentage of eligible patients recruited into the trial was 66.7% (95% CI 52.9%–80.4%; 30/45). Twenty‐nine participants completed the first 6 h of the trial 96.7% (95% CI 82.8%–99.9%, 29/30). Participants were highly supportive of music interventions in the target setting (mean value of 8 (SD ± 1.6) on a scale of 1–10). 95% Confidence Intervals for efficacy included clinically important differences. Specifically, the SBPV was non‐significantly lower in the intervention arm (mean difference − 1.31 mmHg, [95% CI −4.8 to 2.2 mmHg]). Similarly, the adjusted β was non‐significantly lower in the intervention arm for change in pain burden (−3.9 [95% CI −11.4 to 3.7]) and change in anxiety burden (−9.9 [−98.2 to 78.5]).

**Interpretation:**

Our findings support a larger trial of music or sound interventions in hyperacute and acute stroke patients as alternatives to or synergists with pharmacologic management.

## 1 Introduction

Music interventions have been used throughout history for healing with the first documented intervention reported in JAMA in 1914. This article described the anxiolytic effect of the phonograph in the operating room on patients tolerating induction of anesthesia before undergoing the “horrors of surgery.” [1] Music or sound interventions have since been studied in many different medical fields and settings [1, 2, 3, 4]. Recent studies have evaluated the effects of music interventions on hospitalized patients during critical illness and in the peri‐operative cardiac surgery settings [1, 5, 6]. Despite the duration of the studies, trials of music interventions in medicine have had methodological flaws reducing confidence in the literature.

Inherent elements of music or sound stimulate the auditory pathway, sending information from the cochlea to the thalamus with relays to the auditory cortex. Through complex pathways, it is thought that certain inherent elements of music or sound favorably modulate the hypothalamic–pituitary–adrenal axis, limbic, and autonomic nervous system by releasing endorphins and decreasing adrenergic activity [2, 3, 6, 7]. A favorable shift in the autonomic nervous system leads to lower tissue and circulating catecholamines and reduced cortisol levels. Clinical evidence suggests music or sound interventions have beneficial effects on blood pressure, heart rate, cardiopulmonary complications, pain, anxiety, post‐operative infections, and quality of sleep [8, 9, 10, 11, 12, 13, 14]. Even in patients who are not alert and oriented, it is reasonable to believe elements of music or sound may have beneficial effects.

Elevated blood pressure and blood pressure variability occur following stroke and predict worse outcomes. However, the optimal blood pressure goal and management strategy are not known [15, 16, 17, 18, 19]. Guidelines recommend treating extremes of blood pressure while avoiding significant lowering of mean arterial pressure. Indeed, two recent trials showed lowering of blood pressure with pharmacologic agents in hyperacute ischemic stroke worsens outcomes, even if lowering is required to give thrombolytic therapy [20, 21]. In addition to acute blood pressure changes, stroke patients frequently experience pain or anxiety, which can complicate care and adversely affect hemodynamics [22, 23].

There is a paucity of data on the acceptance and efficacy of music or sound interventions during the hyperacute/acute stroke period. Given the potential benefits of music or sound therapy as non‐pharmacological modifiers of acute stroke management endpoints (blood pressure, pain, and anxiety) we designed a pilot feasibility trial in this setting (Music Interventions in Hyperacute and Acute Stroke patients (MIHAS)).

## 2 Methods

This trial was an open‐label parallel group, randomized controlled trial with objective endpoints. The research was conducted at the University of Michigan Health West, a community teaching health system. The hub hospital is a 210‐bed certified comprehensive stroke center with approximately 400 stroke admissions per year. Stroke patients are admitted to the stroke unit or dedicated neurological ICU beds. Patients are managed by an interprofessional team, including vascular neurologists and/or neurocritical care physicians.

### 2.1 Standard Protocol Approvals, Registrations, and Patient Consents

The study was approved by the University of Michigan Health West Institutional Review Board. Study data were managed using Research Electronic Data Capture (REDCap) tools. REDCap is a secure, web‐based application designed to support data capture for research studies [24, 25]. Consent was obtained from the patient or their legally authorized representative. This trial was registered atISRCTN, 13104282 (https://www.isrctn.com/isrctn13104282).

### 2.2 Description of Study Population and Enrollment

Eligible patients (or their legally authorized representative) were approached in the emergency department or on admission to the inpatient ward. Staff not involved in the study implementation used a computer random number generator to generate a randomization sequence, and allocation concealment was obtained by sealed opaque envelopes.

We screened all patients older than 17 years of age with a suspected stroke or transient ischemic attack who could be randomized within 24 h from the onset of symptoms. We excluded patients for the following reasons: (1) expected to die or have care limitations during the hospital stay, (2) patients not expected to complete the first 6 h of the trial period, (3) patients who may require intubation during the study period, including patients prior to thrombectomy, (4) hemodynamically unstable patients who may require vasopressors, (5) patients with severe agitation at enrollment, (6) non‐English speaking patients, and (7) no ability to consent for the patient or legally authorized representative (or refused consent).

### 2.3 Intervention

Excluding the randomization process and the use of the music or sound intervention (in the intervention group), all medical care of participants followed usual, disease‐specific care plans. Participants randomized to the intervention arm had 19 relaxation or pleasant sound stations to ambiently listen to on their in‐room televisions. They also had options to listen to various music genres. Participants were asked to listen ambiently to music or sounds for the first hour of the trial and were encouraged to listen to it frequently for the duration of the trial period; however, there was not a specific time requirement.

### 2.4 Outcomes and Data Collection

The trial period was a minimum of 6 h, with trial termination when the patient was discharged from the stroke unit, or neurological ICU, or after a maximum of 12 h of data collection. The main aims of this pilot trial were to assess the feasibility and acceptance of methods and procedures to inform a larger study on music or sound therapy. The trial is reported consistent with the CONSORT statement and Guidelines for designing and evaluating feasibility pilot studies [26]. The feasibility outcomes were consistent with the SEAR framework [27]. Due to limited funding and utility, we chose not to keep a screening log for this pilot study [28, 29].

### 2.5 Feasibility Outcomes

The primary feasibility outcomes were: (1) the percentage of eligible patients approached for consent who were recruited into the trial and (2) the percentage of enrolled patients who completed the 6‐h trial data collection. Secondary feasibility outcomes included: (3) the percent of eligible patients recruited into the trial, (4) how supportive participants were of using music or sound therapy in the target setting, (5) would they prefer a trial of music or sounds prior to a traditional medication to treat blood pressure, pain (Visual Analog Scale of 1–10) or anxiety (Visual Analog Scale score 0–100), (6) would they prefer to listen to therapy ambiently or via headphones, (7) what types of music/sounds they preferred (Appendix A), and (8) participants in the intervention group were asked “how often did they used the intervention” (Likert five point).

### 2.6 Safety Outcomes

The primary safety outcomes were neurological deterioration defined by an unconfounded increase in NIHSS by at least 4, hemorrhagic complications (hematoma expansion, rebleeding, hemorrhagic transformation type I or II, intraparenchymal hematoma type I or II), or death. Secondary outcomes included agitation or interference with medical care.

### 2.7 Exploratory Clinical Outcomes

Due to the small sample size and limited generalizability, the study was not powered to evaluate the efficacy of the intervention. We do provide exploratory analysis to look for patterns in the data and quantitative analysis of the data to explore 95% confidence intervals (CI). Given that studies targeting the reduction of systolic blood pressure to below a certain goal have given inconsistent results, we defined the main exploratory outcome as the systolic blood pressure variability (SBPV) in the first 6 h of the trial period defined by the standard deviation of the SBP in the treatment arms [30, 31, 32, 33]. Secondary exploratory outcomes included the effect of treatment arm assignment (intention to treat) on the Visual Analog Pain Scale (0–10) and anxiety (Visual Analog Scale 0–100) burden adjusted for baseline pain or anxiety score. Burden was obtained by the summation of hourly values over the first 6 h of the trial period.

### 2.8 Possible Confounders

The therapeutic intensity level of blood pressure control (TILBPC) was measured on an ordinal scale from 0 to 3 as described: 0 (Low) = No Blood Pressure medications needed or if on a continuous infusion the medication was weaned completely off within 4 h, 1 (Mild) = On a continuous antihypertensive drip for > 4 h at a dose of no more than 1/3 of the manufacturers' recommended dosing range or no more than 40 mg combined of labetalol, hydralazine, or equivalent intermittent intravenous medications, 2 (Moderate) = On a continuous antihypertensive drip for > 4 h at a dose of > 1/3rd but less than 2/3rds of the manufacturers' recommended dosing range or no more than 80 mg combined of labetalol, hydralazine, or equivalent intermittent intravenous medications, 3 (High) = Dosing higher than TILBPC levels 0–2 or any use of nipride. For brevity, Table 1 demonstrates baseline demographics and other possible confounding variables.

**TABLE 1:** Baseline demographics by treatment group.

	Intervention (*n* = 16)	Control (*n* = 14)	*p*
Consent: Patient (vs. LAR)	81.3% (13/16)	71.4% (10/14)	0.67
Age (mean [SD])	66.8 (12.8)	66.1 (11.3)	0.86
Gender (Female)	13 (81.3%)	6 (42.9%)	0.03*
Ethnicity			
White	15 (93.7%)	11 (78.6%)	0.45
Black	0 (0%)	2 (14.3%)	
White‐Hispanic	1 (6.3)	1 (7.1)	
Right‐handed	13 (81.3%)	12 (85.7)	1.0
Tabacco use	5 (31.3%)	5 (35.7%)	0.58
Alcohol use disorder	3 (18.8%)	3 (21.4%)	1.0
Qualifying event			
TIA	5 (31.3%)	3 (21.4%)	0.2
Ischemic stroke	7 (43.7%)	11 (78.6%)	
ICH	2 (12.5%)	0 (0%)	
SAH	2 (12.5%)	0 (0%)	
Time from ictus to enrollment			
0–3 h	2 (12.5%)	1 (7.1%)	1.0
3–6 h	6 (37.5%)	5 (35.7%)	
6–9 h	3 (18.8%)	3 (21.4%)	
9–12 h	2 (12.5%)	3 (21.4%)	
12–24 h	3 (18.8%)	2 (14.3%)	
Thrombolytic therapy (in ischemic stroke [*n* = 18])	1/7 (14.3%)	5/11 (45.5%)	0.31
Mechanical thrombectomy (in ischemic stroke (*n* = 18))	0/7 (0%)	4/11 (36.4%)	0.12
NIHSS (in ischemic stroke [*n* = 18]) mean (SD)	4 (2.9)	8 (7.3)	0.1
Charlson comorbidity index (median [IQR])	2 [2.5]	4 [3]	0.17
TILBPC			
0	12 (75%)	11 (78.6%)	1.0
1	4 (25%)	3 (21.4%)	
2	0 (0%)	0 (0%)	
3	0 (0%)	0 (0%)	

Abbreviations: ICH, intracerebral hemorrhage; IQR, interquartile range; LAR, legally authorized representative; NIHSS, National Institute of Health Stroke Scale; SAH, subarachnoid hemorrhage; SD, standard deviation; TIA, transient ischemic attack; TILBPC, therapeutic intensity level of blood pressure control.

* Significant at *p* < 0.05.

### 2.9 Sample Size Determination

No power analysis was performed for this pilot study. A practical sample size of 30 participants was chosen based on budgetary and resource considerations.

### 2.10 Statistical Analysis

Continuous variables were screened for normality using normality plots, histograms, and the Shapiro–wilk test. Parametric data are expressed as mean + standard deviation (SD) and non‐parametric data as median with interquartile range (IQR). The 95% CI for the proportions was obtained using the asymptotic approximation method. Continuous variables with a normal distribution were compared by treatment group with a 2‐sample *t*‐test and non‐normal data with the Wilcoxon rank‐sum test. Linear regression was used to estimate the mean difference and 95% CI for SBPV, baseline pain, and baseline anxiety score by treatment group. Multiple linear regression was used to present the point estimate and 95% CI for change in pain and anxiety burden by treatment group adjusted for baseline scores. Spaghetti plots were used to explore qualitative changes in blood pressure parameters over time. Efficacy analyses were conducted on the intention‐to‐treat population. No corrections for multiple comparisons were performed given the pilot nature of the study. All analyses were performed using SAS 9.4. A *p* < 0.05 was considered statistically significant.

### 2.11 Data Availability Statement

The data that support the findings of this study are available on request from the corresponding author upon reasonable request.

## 3 Results

### 3.1 Feasibility: Trial Enrollment and Completion Rate

**FIGURE 1:**
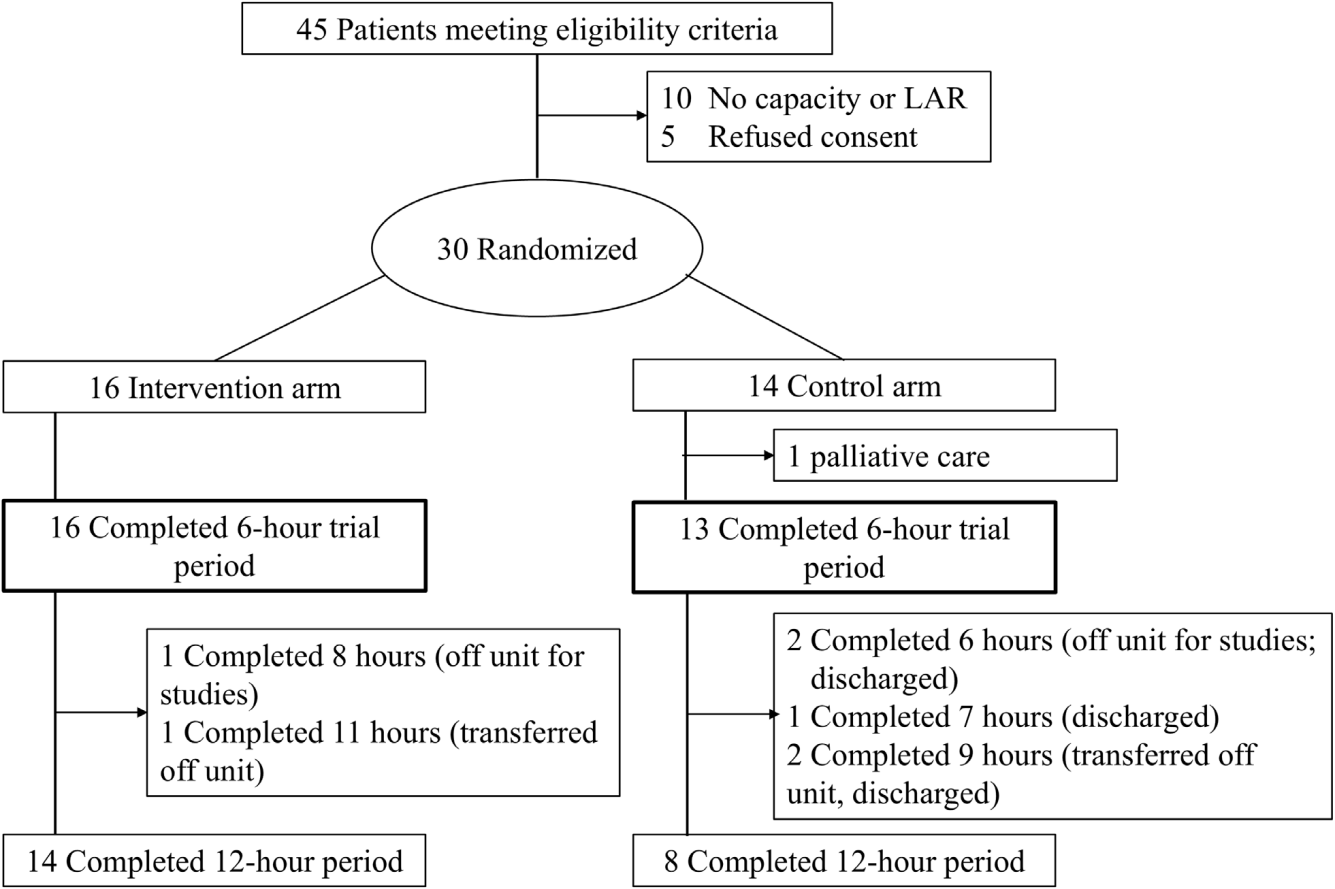
Consort patient flow diagram.

Forty‐five patients were eligible for the trial which enrolled from June 2024 and August through September 15th, 2024. The study was on hold in July 2024 due to a research staffing shortage. Ten patients lacked the capacity to consent and did not have an LAR present within 24 h from ictus. Five patients refused the trial. Hence, the percentage of eligible patients approached for consent who were recruited into the trial was 85.7% (95% CI 75.9%–98%; 30/35) and the percentage of eligible patients recruited into the trial was 66.7% (95% CI 52.9%–80.4%; 30/45). Twenty‐nine participants completed the first 6 h of the trial 96.7% (95% CI 82.8%–99.9%, 29/30), and all had complete data collection. One participant in the control arm suffered a type II parenchymal hematoma immediately after enrollment and was transitioned to palliative care prior to additional data collection. A consort flow diagram can be seen in Figure 1. Importantly, participants were most frequently enrolled 3–6 h from ictus in both groups (Intervention arm 37.5% (6/16) versus Control arm 35.7% (5/14) p‐value 1.0) (Table 1) with the majority of participants in the trial enrolled within 12 h from ictus.

### 3.2 Feasibility: Conceptual and Psychometric Adequacy

When participants were asked how supportive they were of using music or sound therapy in the target setting (within 24 h following a suspected stroke) they reported a mean value of 8 (SD ± 1.6) on a scale of 1–10. Additionally, when participants were asked if they would prefer a trial of music or sounds prior to a traditional medication as the first option to treat elevated blood pressure, 76.7% (23/30; 95% 61.5%–91.8%) responded they would. Similarly, when asked about the treatment of anxiety, 83.3% (25/30; 95% CI 70%–96.7%) responded they would prefer a trial of music or sounds prior to traditional medication. When asked if they would prefer to listen ambiently in the room or via headphones, 83.3% (25/30; 95% CI 70%–96.7%) responded they would prefer ambient noise. All participants were asked which types of music or sounds they would prefer, with the majority preferring pleasant/relaxing sounds over any specific music genre (Appendix—Table A1). In the intervention group, 37.5% (6/16), 31.3% (5/16), and 31.3% (5/16) reported using the intervention always, frequently, or occasionally, respectively. No patient reported rarely or never using the intervention.

### 3.3 Clinical Outcomes

The cohort consisted of 16 patients randomized to the intervention group and 14 to the control group. There were significantly more females in the intervention group (81.3%; 13/16) versus (42.9%; 6/14) the control group (*p*‐value 0.03). All 4 intracranial hemorrhage patients were in the intervention group: 2 ICHs (with ICH scores of 0 and 2) and 2 SAHs (Hunt and Hess grades 2 and 3). Comparatively, there was a trend for more severe ischemic strokes in the control arm (median NIHSS 4 (IQR2.9) in the intervention arm versus median NIHSS 8 (IQR 7.3) in the control arm (*p*‐value 0.1)) which was associated with higher use of thrombolytic therapy and mechanical thrombectomy. However, the therapeutic intensity of blood pressure control was low and did not differ between groups. Table 1 shows the bivariate comparison of the baseline demographics dichotomized by assignment to the intervention or control group. The location of the insult can be seen in Appendix—Table A2.

### 3.4 Safety Outcomes

Table 2 shows the number of complications in each study arm. Complications were rare, with one patient in each arm reporting agitation during the study period. One patient in the control arm suffered a fatal type II intraparenchymal hemorrhage following thrombolytic therapy and withdrew from the study after baseline vital signs were obtained.

**TABLE 2:** Complications by treatment group.

Complications	Intervention (*n* = 16)	Control (*n* = 14)
Agitation	1 (6.3%)	1 (7.1%)
Interference with care	0 (0%)	0 (0%)
Neurological deterioration	0 (0%)	1 (7.1%)
Hemorrhagic complications	0 (0%)	1 (7.1%)
Death	0 (0%)	1 (7.1%)

### 3.5 Efficacy Outcomes: SBPV and Blood Pressure Measurements Overtime

**FIGURE 2:**
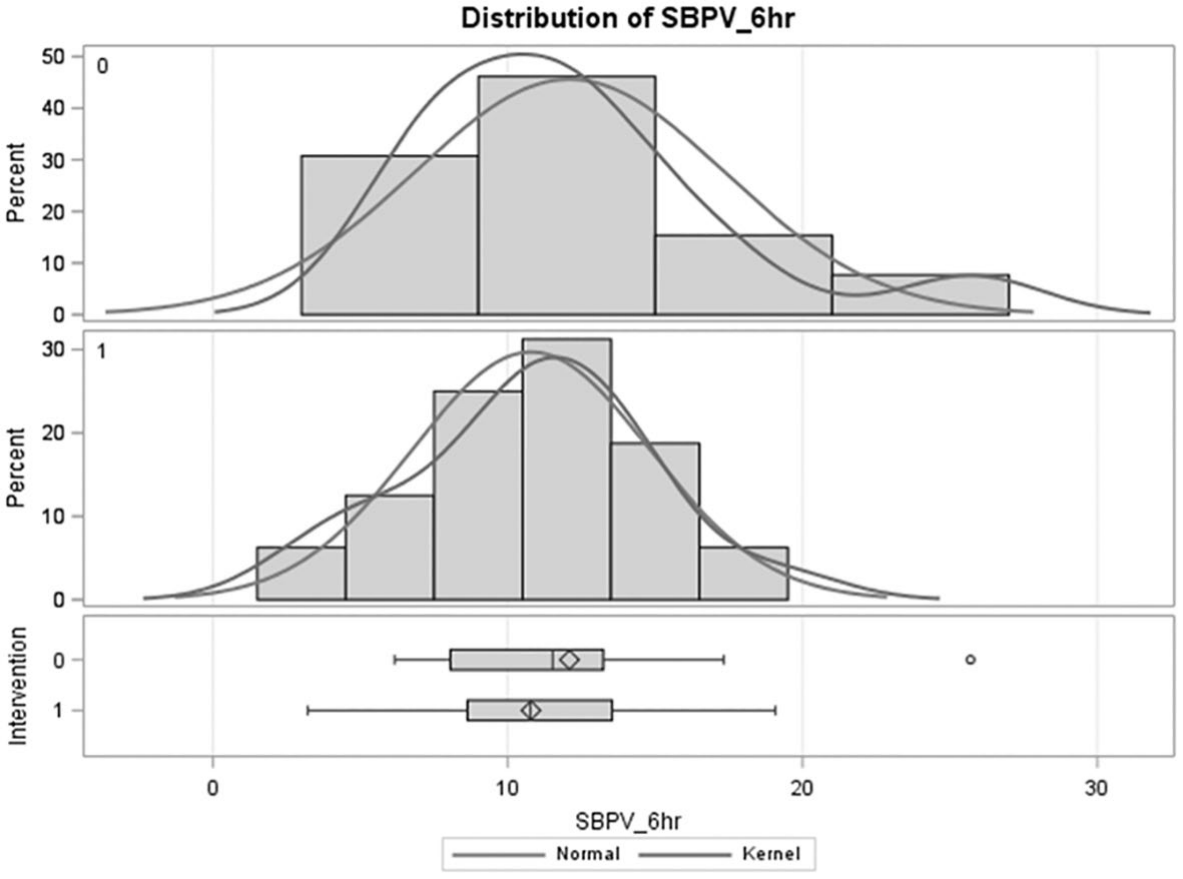
Distribution of SBPV by treatment group. Treatment 0 = control, treatment 1 = intervention.

**FIGURE 3:**
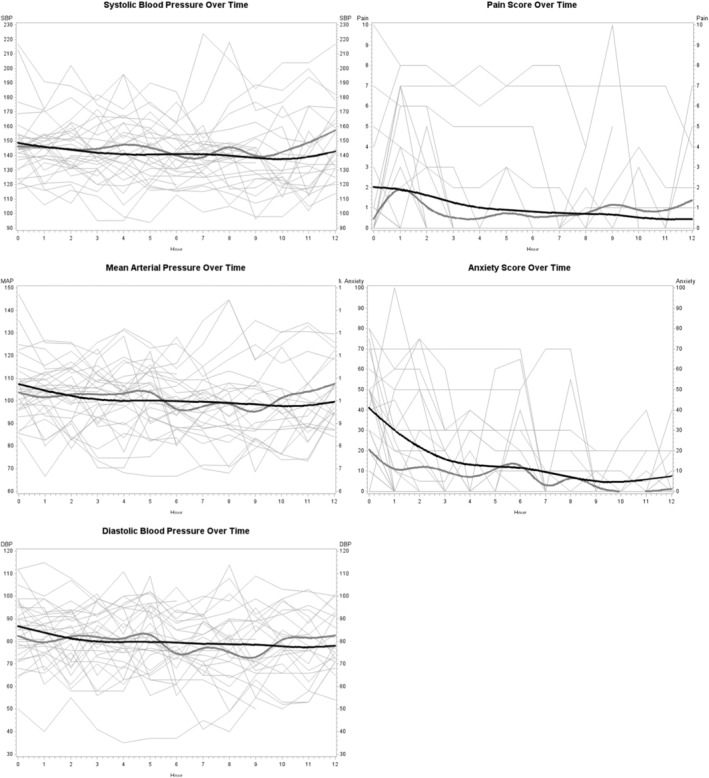
Spaghetti plots of blood pressure parameters, pain, and anxiety over time. The dark black lines are mean values in the intervention arm. The grey lines are the mean in the control arm. Visually less variability and favorable trends are seen in all parameters in the intervention time.

The SBPV (defined as the standard deviation of the SBP) at 6 h was non‐significantly lower in the intervention arm (10.8 mmHg) than in the control arm (12.1 mmHg) (−1.31 (95% CI −4.8 to 2.2 mmHg; *p* = 0.45)) (Figure 2). Spaghetti plots of the SBP, MAP, and DBP over time can be seen in Figure 3. Qualitatively, these graphs support less variability and trends towards lower blood pressure parameters measured over time.

### 3.6 Efficacy Outcome: Pain and Anxiety

Baseline anxiety was significantly higher in the intervention than the control arm (43.4 vs. 20.7, MD 22.7; 95% CI 3.9–41.6), while baseline pain, total pain burden, and total anxiety burden were also all numerically higher in the intervention arm. When adjusting for the baseline pain or anxiety score, patients in the intervention arm had a non‐significant trend to lower pain (β −3.9 (95% CI −11.4 to 3.7)) and anxiety burden (β −9.9 (95% CI −98.2 to 78.5.4)) (Table 3).

**TABLE 3:** Unadjusted and adjusted analysis of pain and anxiety by treatment group.

Unadjusted	Intervention (*n* = 16)	Control (*n* = 13)	MD (95% CI)
Baseline pain (Scale 1–10)	1.9 (±3)	0.43 (±1.1)	1.5 (−0.28 to 3.2)
Baseline anxiety (Scale 0–100)	43.4 (±20.1)	20.7 (±21.3)	22.7 (3.9 to 41.6)*
Pain burden (total over 6 h)	7.8 (±13.3)	4.8 (±11.6)	2.9 (06.7 to 12.5)
Anxiety burden (total over 6 h)	104.1 (±120.9)	65 (±114.2)	39.1 (−51.3 to 129.5)

Adjusted for baseline score		*β* (95% CI)
Pain burden	Ref control arm	−3.9 (−11.4 to 3.7)
Anxiety burden	Ref control arm	−9.9 (−98.2 to 78.5)

Abbreviations: CI, confidence interval; MD, mean difference.

* Significant at *p* < 0.05.

## 4 Discussion

Overall, in a moderate‐volume comprehensive stroke center, this pilot trial demonstrated high enrollment in the hyperacute stroke period over a short enrollment period. A high completion rate by participants and high compliance with data collection by research staff was observed. Additionally, participants and/or their LARs reported high support for music or sound therapy, with the majority preferring a trial of music or sound prior to a traditional pharmacologic medication. Though consenting to be in the trial may bias answers to supportive questions toward positive responses, high agreement to be in the trial among eligible patients is congruent with these findings. Hence, our findings strongly support the feasibility of a larger trial of music and/or sound therapy in acute stroke patients.

The high percentage of participants or LARs who stated they would choose a trial of music or sound prior to a traditional medication deserves further comment. Standard of care for elevation of blood pressure following stroke advocates for rapid initiation of intravenous medications, with a strong push for continuous infusions to control variations in parameters [15, 16, 17, 18, 30, 32, 34, 35]. Additionally, despite non‐pharmacological options for pain or anxiety, these are also most frequently treated with intravenous pharmacologic agents in the acute stroke setting. Unfortunately, all these medications have potentially significant dose‐related adverse central nervous system effects and may increase organ failure, the duration of mechanical ventilation, and ICU length of stay [1, 36]. Additionally, medical and “big pharma” mistrust has increased following the COVID‐19 pandemic, making some patients less likely to accept some standard therapies. Hence, music or sound interventions may be low‐cost alternatives or synergistic interventions to traditional medications that have the potential to reduce adverse events and are highly accepted by patients.

The most valid measurement and clinically important difference (CID) of SBPV has not been clearly defined. However, most studies use the SD of the mean systolic blood pressure as the primary measure [16, 30, 31, 32, 33]. Clinically important differences, defined by functional outcome (such as dichotomized mRS or utility‐weighted mRS) or surrogate outcomes (such as reperfusion, infarct or hematoma growth) have been seen with between‐group differences of 2–7 mmHg.

Though our study was not powered to look for any significant differences in clinical efficacy, the 95% CI for the SBPV likely includes CIDs, as does the 95% CI for pain and anxiety. Qualitative spaghetti plots of these variables also show favorable directional trends with less variation.

Specific limitations of our study deserve mention, keeping in mind the pilot feasibility aim of the trial. First, non‐invasive hourly measurements of vital signs are not the most sensitive measure of changes in blood pressure parameters over time, as they may overlook critical fluctuations. Future trials with continuous measures would be ideal. Second, the use and intensity of blood pressure medications could have confounded our findings. However, the TILPBC was low and similar on both intervention arms. Third, the sample size was small, which limits statistical power and generalizability. This also limited the ability to look at long‐term functional outcomes and other patient‐centered outcomes such as post‐traumatic stress disorder, which can affect quality of life following stroke. Fourth, the absence of blinding may have influenced bias in the reporting of pain and anxiety. However, given that the risk and cost of music interventions are low, a participant's belief that music interventions will reduce pain or anxiety (placebo effect) is a positive response if it leads to a reduction in these symptoms. Lastly, the dose (how often the music or sound intervention was used) of the intervention was not controlled after the first hour of the study and was only monitored with an ordinal Likert scale at the end of the trial period.

## 5 Conclusion

Our trial demonstrated that a larger trial of music or sound therapy is feasible in patients suspected of acute stroke and would likely be well accepted by patients and LARs. The intervention appears safe, with quantitative and qualitative outcome data suggesting the potential for CIDs in acute stroke management endpoints.

## Author Contributions

Jeffrey Fletcher contributed to the conception, design, data collection, statistical analysis, and writing and revision of the manuscript. Augusto Elias, Ronald Grifka, Joan Westendorp, Allison Edberg, Jacquie Knott, Elizabeth Martin, and Fazeel Siddiqui all contributed to the conception, data collection, writing, and revision of the manuscript. All authors approved the final version of the manuscript and agreed to be accountable for the work.

## Conflicts of Interest

The authors declare no conflicts of interest.

## Data Availability

The data that support the findings of this study are available on request from the corresponding author. The data are not publicly available due to privacy or ethical restrictions.

## Appendix A

**TABLE A1:** Type of music/sound preferences choosen.

Type of Music/Sound	Participants (30)
Pleasant/Relaxing sounds	28
Classical	3
Gospel	1
Christian	3
Country	7
Bluegrass	1
Rock	2
Jazz	0
Pop	0
Blues	1
40s	0
50s	0
60s	0
70s	0
80s	0
90s	0
Other	1

**TABLE A2:** Location of insult.

Location of insult	Intervention (*n* = 16)	Control (*n* = 14)
Right cortex	3	4
Right subcortical/Basal ganglia	3	0
Right brainstem	3	1
Right cerebellum	0	1
Left cortex	2	5
Left subcortical/Basal ganglia	2	0
Left brainstem	0	1
Left cerebellum	2	2
Bilateral/diffuse	1	0
